# A Scientific Cognitive-Behavioral Model of Tinnitus: Novel Conceptualizations of Tinnitus Distress

**DOI:** 10.3389/fneur.2014.00196

**Published:** 2014-10-06

**Authors:** Laurence McKenna, Lucy Handscomb, Derek J. Hoare, Deborah A. Hall

**Affiliations:** ^1^Royal National Throat Nose and Ear Hospital, University College Hospitals, London, UK; ^2^Nottingham Hearing Biomedical Research Unit, National Institute for Health Research (NIHR), University of Nottingham, Nottingham, UK; ^3^Otology and Hearing Group, Division of Clinical Neuroscience, School of Medicine, University of Nottingham, Nottingham, UK; ^4^UCL Ear Institute, University College London, London, UK

**Keywords:** tinnitus, belief, distorted perception, negative thoughts, safety behavior, selective attention

## Abstract

The importance of psychological factors in tinnitus distress has been formally recognized for almost three decades. The psychological understanding of why tinnitus can be a distressing condition posits that it becomes problematic when it acquires an emotive significance through cognitive processes. Principle therapeutic efforts are directed at reducing or removing the cognitive (and behavioral) obstacles to habituation. Here, the evidence relevant to a new psychological model of tinnitus is critically reviewed. The model posits that patients’ interpretations of tinnitus and the changes in behavior that result are given a central role in creating and maintaining distress. The importance of selective attention and the possibility that this leads to distorted perception of tinnitus is highlighted. From this body of evidence, we propose a coherent cognitive-behavioral model of tinnitus distress that is more in keeping with contemporary psychological theories of clinical problems (particularly that of insomnia) and which postulates a number of behavioral processes that are seen as cognitively mediated. This new model provides testable hypotheses to guide future research to unravel the complex mechanisms underpinning tinnitus distress. It is also well suited to define individual symptomatology and to provide a framework for the delivery of cognitive-behavioral therapy.

## Introduction

Tinnitus is defined as the perception of sound in the absence of auditory or electrical stimulation. It is one of the most common somatic symptoms to affect humanity, with a point prevalence of 10.1% of the population (1). The number of people who are distressed by tinnitus is, however, much smaller. Just under half of people with tinnitus report that it has at least some effect on their lives (2) and one tenth describe tinnitus as having a major negative effect on their lives (1). Those who do suffer with tinnitus complain of anxiety, depression, insomnia, auditory perceptual dysfunction, and concentration problems (3). Understanding why some people suffer and others do not is one of the major challenges in tinnitus research and clinical practice. Many patients suggest that their distress is an inevitable consequence of the psychophysical characteristics of their tinnitus. While this explanation has an intuitive appeal, there is, however, little evidence to indicate a relationship between distress and the psychophysical characteristics of tinnitus, such as loudness estimated by matching to an external tone [e.g., Ref. (4, 5)]. Self-report characteristics, such as loudness rated on a visual analog scale (VAS), correlate only moderately with measures of self-reported distress, and weakly with psychoacoustic measures of loudness, so quite what such scales are measuring is questionable (6).

The role of psychological factors in determining distress in patients with tinnitus has long been recognized and remains a central theme in researchers’ and clinicians’ views of tinnitus [e.g., Ref. (7–10)]. The mechanisms by which psychological factors operate to produce or alleviate tinnitus-related distress have been much debated [e.g., Ref. (11, 12)]. With respect to the generation of tinnitus, the neurophysiological model of tinnitus (13–15) posits that tinnitus becomes problematic when there is a temporal association with an event that evokes a negative emotional state. Here, tinnitus is conceptualized within the classical conditioning paradigm and so this perspective asserts that the unconscious conditioning is more important than the conscious evaluation of tinnitus (11). In other words, the psychological (cognitive) component is not critical to the understanding of tinnitus. The therapeutic approach that emerges from this conceptualization is Tinnitus Retraining Therapy (TRT). It is suggested that the key therapeutic process is one of the passive extinction of the conditioned response. It is proposed that this is achieved first by educating the patient about the benign nature of tinnitus and second by using external sound to decrease the perceptual salience of the tinnitus, so reducing autonomic nervous system activity. TRT does not emphasize a need to change the patient’s cognitions because these are not held to modulate the conditioned reflex (15).

### An influential psychological perspective

In contrast, the psychological model of tinnitus considers cognitive processes to play a primary role in the tinnitus experience and in its clinical management. One of the most influential psychological perspectives has been the habituation model (7). In this model, high levels of arousal or stress are proposed to reduce the ability to filter out and ignore tinnitus-related information (“dishabituation”). Thus, in a reciprocal feedback loop, orienting to tinnitus may in turn increase arousal and hence further diminish habituation. Although Hallam et al. (7) did not offer precise details about the particular cognitive processes that might be involved in tinnitus detection and distress, their model has been the main inspiration for clinical psychologists working with patients with tinnitus. According to this perspective, tolerance to tinnitus can be facilitated by reducing levels of autonomic nervous system arousal, changing the emotional meaning of the tinnitus, and reducing other stresses. To date, therapeutic approaches have typically involved relaxation training to reduce arousal and cognitive-behavioral therapy (CBT) to identify and change the emotional meaning of tinnitus (16, 17).

### Elaborations to Hallam’s habituation model

There have been subsequent elaborations and refinements to Hallam et al.’s (7) original conceptualization of the psychological model. One variation proposed a role for operant conditioning mechanisms, giving rise to avoidance behavior as a further correlate of tinnitus disability. The full text of this model was published in German (18), but a summary has been provided in English in a later publication (19). The model also highlights the physiological contributions of central auditory dysfunction in tinnitus etiology, with subsequent adaptation as a psychologically determined process (hence the name “psychophysiological model”). The sequential perspective on the process is reflected in the recommended clinical approach, which starts with education, advice for self-help, and relaxation, followed by CBT only for those patients still reporting substantial distress. The serial nature of the model, however, is perhaps its major theoretical limitation. There is no clear empirical evidence that the tinnitus experience should evolve in this particular prescribed serial manner.

Another variation has emphasized the importance of the signal-to-noise properties in influencing the changing state of the tinnitus experience (2). Andersson suggested that tinnitus has an impact on cognitive functioning in the way that ambient environmental noise might do and that this serves as the basis for conditioned emotional reactions. When the disruption in cognitive functioning is noticed, the person attends more to the tinnitus. Selective attention and monitoring enhance the perception of tinnitus potentially leading to the subjective experience and self-report of an increase in loudness (see Selective Attention and Monitoring and Distorted Perception). Andersson et al.’s (2) arguments imply a role for negative thoughts but he states: “Fear of a brain tumor is most likely not sufficient to explain patients’ emotional responses to tinnitus, and neither is fear of becoming deaf” (p. 199). Cognition is, therefore, seen as playing a role but not necessarily a central one in tinnitus distress. The clinical implications of the model are unclear, but there would seem to be as large a role for the manipulation of environmental sound levels (e.g., using external sound to decrease the perceptual salience of the tinnitus) as there would for the manipulation of cognitions.

### Some shortcomings of existing psychological models

Despite specific recommendations for CBT, the models of tinnitus proposed by psychologists (2, 7, 18, 19) are not strictly cognitive behavioral. We would also posit that they are more akin to conceptual frameworks rather than models *per se*. This section briefly expands on these two points.

The key defining characteristic of a cognitive-behavioral approach is the assumption that behaviors are cognitively mediated and so can be changed or altered by a process of conscious cognitive enquiry. We note that although Hallam et al. (7) suggested that an orienting response might lead to an interruption in behavior and this, in turn, would increase arousal, there is no assertion of the need for cognitive mediation of the behavior *per se*. The same argument is true for Kröner-Herwig et al. (19). As in Jastreboff’s classical conditioning model, operant conditioning is not a strictly cognitively mediated process. Although Andersson’s viewpoint emphasizes conscious voluntary processing, it remains unclear how the disruption of attention is separate from the influence of cognitions (personal communication). Thus, none of these models explicitly set out the mechanism by which behavior might be cognitively mediated. While it is highly likely that automatic behavioral processes operate in tinnitus perception, ignoring the cognitive motivation for behavior and the impact of behavioral changes on cognition leads to a conceptual shortfall and risks missing therapeutic possibilities. Current psychological conceptualizations also suggest that changes to both automatic and deliberate behavioral processes require conscious cognitive change (20).

For a model to be regarded as scientific, there must be some way of testing whether it is false and so a model should at least provide testable hypotheses about a particular process or mechanism (21). Previous work provides a useful set of heuristics or a broad conceptual framework, but remains a description of a collection of factors, rather than implicating any specific mechanisms. In his general questioning of the psychological approach, McKenna (12) highlighted the need for a more highly structured cognitive-behavioral model calling for a careful description of all of the cognitive-behavioral processes surrounding tinnitus and their interrelationship, including their relationship to tinnitus detection. A model set out in such terms would permit a number of testable hypotheses to be developed that might provide the psychological model with a firmer scientific basis.

## A Novel Cognitive-Behavioral Model of Tinnitus

**Figure 1:**
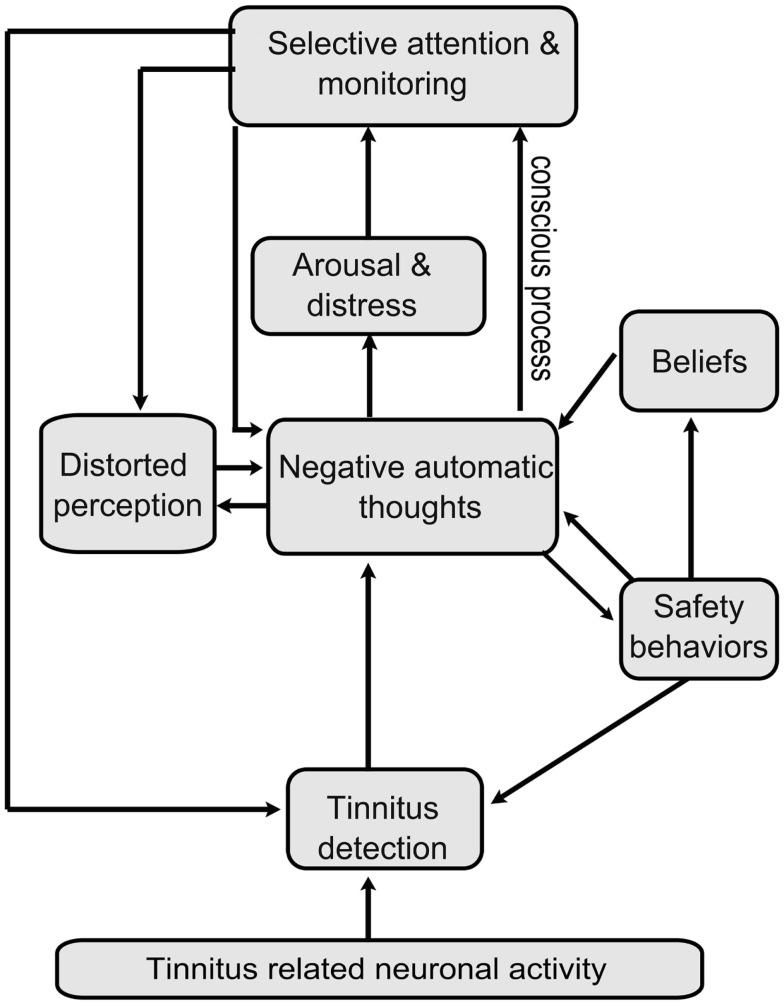
**A cognitive model of tinnitus distress**. Representation of the novel cognitive-behavioral model of tinnitus distress. Tinnitus provokes distress when a person holds overly negatively thoughts about it. These negative thoughts provoke arousal and emotional distress and motivate maintaining factors such as selective attention, monitoring, and counterproductive safety behaviors. These processes result in the patient overestimating the intensity and complexity of tinnitus, i.e., patients gain a distorted perception of tinnitus. Distorted perception is also fueled by overly negative thoughts of tinnitus. A number of feedback loops are involved: selective attention and monitoring leading to greater detection of tinnitus, to further negative appraisal; distorted perception of tinnitus leads to further negative appraisal and we tentatively hypothesize that negative appraisal contributes to distorted perception. In many cases, safety behaviors adopted to cope with the perceived threat inadvertently maintain or exacerbate existing worry and in many cases also directly alter the detection of tinnitus, e.g., by manipulating environmental sounds. Beliefs about tinnitus also fuel negative thoughts. Such beliefs are based on experience of tinnitus in others or derive from general beliefs about health, the self, or the world. Both conscious and involuntary processes are involved but the model emphasizes conscious processes as these constitute the main therapeutic targets.

McKenna (12) raised the question about whether the work of clinical psychologists was located within a specific domain of tinnitus or, more likely, within a model of emotional distress. In recent years, a family of cognitive-behavioral models has been developed to account for problems such as anxiety (22, 23), chronic pain (24, 25), and insomnia (26). These models propose that people experience persistent anxiety about such difficulties because they misinterpret the symptoms, or variations in them, or information regarding them, as evidence of serious physical illness. This is experienced by the person as catastrophic appraisals about their (ill) health. Distress persists because various processes (especially behavioral changes) maintain the overly negative interpretations from which the anxiety results. As these processes are motivated by threat beliefs, vicious circles form and the distress is maintained. Harvey (26) proposed a circuit between negative cognitive activity, beliefs, and avoidance behaviors in the maintenance of insomnia. This model provides inspiration for our model of tinnitus distress (Figure 1).

Our novel conceptualization of tinnitus is consistent with recent research and theorizing in these other areas of emotional distress (23, 26). Specifically, the model shown in Figure 1 asserts that, whatever the original cause of the tinnitus, cognitive-behavioral processes contribute to the development and maintenance of tinnitus distress starting with intrusive negative thoughts about tinnitus. For example, an interruption in behavior, a failure to complete an everyday task, or a spouse’s reaction are all considered to have their effect because of the patient’s *interpretation* of them. The application of the general model of emotional distress to tinnitus is perhaps historically supported more by clinical experience than by empirical evidence. In the remainder of this article, we, therefore, present and critically evaluate the different components of the model with reference to the available scientific evidence.

## Evaluating the Scientific Evidence

Each of the different components of the model is reviewed sequentially with reference to the available scientific evidence with respect to their role in the maintenance of tinnitus and tinnitus distress, to the effect of interventions targeting that component of the model, and to their connections with other parts of the model.

### Excessive negatively toned cognitive activity

Theory underpinning CBT sees negative thoughts as essential to the development and maintenance of a negative emotional state, whether that state is depression, anxiety, or a more specific condition such as social phobia or health anxiety (27). CBT works to a principle that there is no immediate link between events and emotions. Rather, an event triggers thoughts and these thoughts, in turn, give rise to the subsequent emotional state. Any given event may trigger different thoughts in different people. For one person, a creaking sound in the night may trigger the thought: “someone is trying to break in!” and could lead to anxiety. For someone else, the same creaking sound may trigger the thought: “ah, here comes my lovely cat!” and lead to pleasurable sensations. It is also the case that a given individual may experience different thoughts about the same event at different times. This point is illustrated by the common experience of worry while lying awake in the middle of the night. People often report that their thinking is much more negative at that time than in the middle of the day. Many psychological disorders are characterized by a tendency toward negative automatic thoughts, that is, thoughts that are negative in their content and which “pop in” to the thinker’s mind without a deliberate decision to think them (27). Another characteristic of the CBT conceptualization of problems is that the thoughts associated with distressing emotional states are considered *overly* negative. While the events confronting a person may be widely acknowledged as difficult, distress arises, or persists, because the person interprets things as worse than they actually are.

#### Negative thinking in tinnitus

Our suggestion is that the process of distress starts with patients with tinnitus experiencing intrusive *overly* negative thoughts when they detect tinnitus. Patients with tinnitus make negative appraisals about the unnaturalness of the tinnitus, about it escalating, about it interfering with normal activity, about an inability to cope, and possible psychiatric consequences. In this section, we review the research evidence for this component of the model. The findings from questionnaire studies of tinnitus complaint add support to the inclusion of this variable within the model.

Wilson and Henry (28) developed a Tinnitus Cognitions Questionnaire (TCQ) to assess positive (13 statements) and negative (13 statements) cognitions associated with tinnitus. Two hundred people with tinnitus were asked to indicate how frequently they had the thoughts listed. In a subsequent psychometric validation, the most commonly endorsed statements were “why me? Why do I have to suffer this horrible noise?” “I can’t enjoy what I’m doing because of the noise,” and “the noise will drive me crazy.” Overall, the study sample primarily endorsed items indicating that they engaged in cognitions that reflect despair, persecution, hopelessness, loss of enjoyment, a desire for peace and quiet, and beliefs that others do not understand the severity of the noise. There was no correlation between the positive and negative subscales, suggesting that the absence of positive thoughts does not necessarily imply the presence of negative thoughts. The highest endorsement rates were all for negative statements. The authors found very good internal consistency and adequate test–retest reliability. A moderate correlation has been shown between scores on the TCQ and the Tinnitus Reaction Questionnaire (TRQ) (29), a measure of tinnitus distress, indicating that tinnitus distress and tinnitus cognitions are related although not identical constructs. However, the instrument has been little used outside Australia and so its validity across cultural boundaries has not been demonstrated.

Other questionnaires, such as the Tinnitus Questionnaire (TQ) (30), the Tinnitus Handicap Questionnaire (THQ) (31), and the TRQ assess distress or handicap associated with tinnitus reflect both cognitive and behavioral aspects of tinnitus complaint. Cognitive items within these questionnaires include statements, such as “It is unfair that I have to suffer with my noises” and “It will be dreadful if the noises never go away.” The fact that people with tinnitus endorse such statements further supports the idea that patients with tinnitus experience negative thoughts about their tinnitus. Factor analyses of these questionnaires suggest that there are a number of components to tinnitus complaint. A common theme is emotional distress related to tinnitus, which is associated with resentment of the persistence of the noises, a wish to escape them, and worries about health and sanity. Other common themes are complaints about the effects of tinnitus on hearing, sleep disturbance, and other somatic symptoms. The “emotional disturbance” factor, which is largely made up of such cognitive items, accounts for the largest portion of the variance in TQ (30). This supports the idea that cognitive factors are a central element in tinnitus distress.

A small number of studies have used questionnaire tools to investigate specifically catastrophic thinking in patients with tinnitus, that is, thinking about tinnitus in an exaggeratedly negative way. Cima et al. (32) developed a Tinnitus Catastrophizing Scale (TCS), based upon a similar pain questionnaire (33). The TCS is a 13-item, single factor questionnaire. It asks patients to indicate on a five-point Likert scale how frequently they have certain thoughts. Examples of items include “I worry all the time about whether the tinnitus will end” and “I become afraid the tinnitus will get worse.” In their psychometric validation, the authors showed excellent internal consistency. Higher, more catastrophic, scores negatively correlated with quality of life measured with the SF36 (34) and positively correlated with tinnitus severity measured with the TQ. Weise et al. (35), using a different measure of catastrophizing, also found a link between this, poorer coping and more frequent medical visits. These findings also support the conclusion that *overly* negative thinking about tinnitus is an important component of tinnitus distress.

#### Interventions on negative thinking in tinnitus

Cognitive-behavioral therapy seeks to disrupt cycles that cause distress (36). Clinical interventions usually, therefore, include activities designed to modify negative thoughts. Most CBT intervention studies have used outcome measures of tinnitus distress rather than of negative thoughts, and so any specific effect of intervention on negative thinking is not well evidence based. There are, however, a small number of informative studies. A study by Henry and Wilson (37) demonstrated that “cognitive skills training” significantly reduced scores on a pre-publication version of the TCQ compared to control (group education or waiting list). A more recent intervention program involving CBT (38) was also found to be associated with a reduction in catastrophic thinking post-therapy. A larger study by Cima et al. (39) found that a specialized program of CBT, that included “cognitive restructuring” exercises, significantly reduced scores on the TCS compared to a “standard tinnitus care” control measured at 8 months after the start of therapy. The findings from these studies are broadly supportive of this component of the model, but further research is warranted.

#### Connections to other components of the tinnitus model

The scientific model (Figure 1) proposes that negative automatic thoughts give rise to arousal and emotional distress. Some evidence for this is provided by Budd and Pugh (40) who, as part of an investigation into beliefs and coping styles, identified catastrophic thoughts such as “my tinnitus will lead to a nervous breakdown” as important determinants of maladaptive coping and depression. A possible caveat, however, is that because behavioral strategies (such as avoiding activities) were assessed by the same measure, it is difficult to judge the relative contributions of the various components of maladaptive coping to distress. Wilson et al. (28) also reported a moderate correlation between the negative subscale of their TCQ and the Beck Depression Inventory (BDI) supportive of the claim for a link between negative thinking and emotional distress (in this case, depressed mood). Weise et al. (35) also found an association between catastrophic thinking and depressive symptoms and argued that catastrophizing in the early stages of the tinnitus experience has a pivotal role in determining long-term distress.

Catastrophizing has also been associated with fearful beliefs, increased attention toward tinnitus (32), and high self-reported loudness ratings (35). This lends some support to the proposed link between both beliefs and distorted perception and negative automatic thoughts about tinnitus and between negative thoughts and selective attention (the model proposed here suggests that the latter link is mediated by arousal and distress, but this remains uninvestigated).

### Arousal and distress

In keeping with other cognitive-behavioral models, it is suggested that negative thoughts trigger autonomic arousal and emotional distress.

#### Autonomic arousal in tinnitus

Patients with tinnitus often complain of feeling tense or “on edge,” but the extent to which autonomic arousal contributes to a negative tinnitus experience is far from clear.

A number of studies have examined biological and physiological indicators of autonomic arousal. Hébert et al. (41) found that cortisol measures from people with bothersome tinnitus across a 1-week period exceeded those of controls or people with non-bothersome tinnitus, indicating greater autonomic arousal. Hébert and Lupien (42) found a delayed and blunted cortisol response in tinnitus subjects presented with a stress-inducing task. They suggested that chronically elevated baseline levels of cortisol in distressed patients with tinnitus leads to an inefficient cortisol response in the face of specific stresses. These preliminary findings indicate that arousal may be a factor that differs between people with bothersome tinnitus, non-bothersome tinnitus, and no tinnitus.

Electromyography provides a different indicator of stress levels. Patients with tinnitus report a higher level of muscle tension (in face, jaw, and shoulders) than healthy controls (4, 43, 44). Rief et al. (45) reported reductions in muscle activity following psychophysiological therapy for tinnitus, implying that tinnitus distress is related to higher arousal levels.

It is noted that self-reported stress is not always associated with physiological indicators. Hébert and Lupien (42) failed to find a difference in self-rated stress levels between patients with tinnitus and controls after completing stress-inducing tasks.

Heinecke et al. (46) reported a mixed picture, higher subjective reports of strain among patients with tinnitus subjected to laboratory stress than among controls but no differences in electromyography or skin conductance measures. The authors suggest that patients may have overestimated the stress-inducing effects of tinnitus and it seems possible that some sort of cognitive distortion is taking place; people with tinnitus might be more likely to think negatively about fairly small changes in arousal. Some support for this idea comes from Hesser and Andersson (47) who investigated anxiety and anxiety sensitivity (a fear of bodily sensations associated with anxious arousal, which is common in individuals who have panic attacks) (48). Using broad questions about tinnitus distress and an anxiety sensitivity index, a multiple regression analysis revealed that anxiety sensitivity was a significant predictor of tinnitus distress, even when anxiety itself was controlled. It should be noted, however, that high levels of trait anxiety have also been found among patients with tinnitus (49, 50).

#### Emotional distress in tinnitus

Although the term “emotional distress” may encompass a wide range of feelings, most studies have limited their investigations to anxiety and depression. Four general themes have been addressed in the literature and these are discussed below.

##### Population characteristics of tinnitus and self-reported mental health problems

The first theme considers whether those in the general population with tinnitus are more likely to report symptoms of anxiety and/or depression than people without tinnitus. A number of large-scale epidemiological studies of the general population have reported increased likelihood of depressive symptoms (51–53) and generalized anxiety (54) among people with tinnitus. A recent study in the UK found an association between bothersome tinnitus and considering oneself to be a “worrier” and a tendency to feel miserable (55). These studies point to an association between tinnitus and emotional distress.

##### Mental health conditions among patients with tinnitus

The second theme considers what proportion of tinnitus clinic patients meet diagnostic or screening criteria for a mental health condition. Using the Structural Clinical Interview for DSM Disorders (SCID, which derives from the American Psychiatric association’s Diagnostic and Statistical Manual of Mental Disorders), Marciano et al. (56) found that 77% of new patients at an Italian tinnitus clinic had a psychiatric disorder during their lifetime. Anxiety and depression were the most common diagnoses. Comparable prevalence rates of lifetime anxiety and depression using SCID were reported by Zoger et al. (57). These authors also reported on disorders present at the time of the interview; 55% of patients were found to have a current mental disorder (45% anxiety, 39% depression). Goebel and Floetzinger (58) reported a 69% prevalence of current psychiatric disorder among patients at a specialist tinnitus center. Depression was the most common disorder, affecting 57%, with anxiety affecting 43.5%, some with both diagnoses. These similar patterns of co-morbidity were obtained despite using an alternative diagnostic interview schedule based on the World Health Organization International Classification of Disease.

Psychiatric diagnoses require specialist training and so many studies have relied on simpler screening tools designed to flag up a suspected problem. Only current, not lifetime, disorders are considered. One of the most widely used tools is the Hospital Anxiety and Depression Scale (HADS) (59). Applying a cutoff score of 11 out of 21 for “probable” mental health problems, Zoger et al. (60) found the prevalence of anxiety to be 12% and depression to be 18% in a sample of 98 tinnitus clinic patients. Using the more conservative cutoff score of 8 out of 21, Bartels et al. (61) reported that, in a tinnitus clinic patient population of 265, 49% had “possible” anxiety, while 49% also had “possible” depression and 39% had both.

##### Typical scores on emotional distress measures

The third theme considers whether people with tinnitus generally have high scores on measures of emotional distress. While there is a broad spread of individual scores, cohort studies of the general public (62–64) and clinical trials of patients (39, 65) have both demonstrated mean scores on the HADS that fall below the clinically meaningful cut offs [see also Ref. (42, 66–68)]. This suggests that emotional distress is far from being an inevitable part of tinnitus experience, leading to the question discussed below.

##### Association between tinnitus severity and measures of emotional distress

The fourth theme considers whether those with tinnitus which they rate as more severe, are likely to be more emotionally distressed. If the predictions of the cognitive model are correct and emotional distress is part of a more negative tinnitus experience, it should be higher among those patients who are finding their tinnitus more troublesome. The weight of evidence seems to support the idea that people experiencing general anxiety and depression are more likely to find their tinnitus highly distressing, but the direction of causality has not been clearly established. A few cross-sectional studies have found a positive correlation between measures of tinnitus distress and emotional distress. For example, Zoger et al. (10) reported significant correlations between tinnitus severity and the two subscale scores of the HADS [see also Ref. (49, 69)]. Milerova et al. (70) found evidence for a link between tinnitus distresses measured on the TQ and the Tinnitus Handicap Inventory (THI) (71) and the depression subscale of the Symptom Checklist-90-R (72), but not the anxiety subscale. In contrast to this latter observation, a longitudinal cohort study of new patients in the clinic showed that anxiety at tinnitus onset predicted severe tinnitus distress 6 months later (73).

#### Interventions on autonomic arousal and emotional distress in tinnitus

Relaxation training seeks to reduce autonomic arousal and is often prescribed in combination with other interventions. Only a small number of randomized controlled trials have assessed approaches to therapy based primarily on physiological relaxation (16). Results are mixed, and the quality of evidence is low to moderate. Further clinical trials are warranted.

If the connections between components in the model are correct, then interventions that seek to reduce general emotional distress should be effective in reducing tinnitus-related distress. Meta-analyses of the effects of CBT on tinnitus-related distress have been carried out. Andersson and Lyttkens (74) analyzed 24 studies (*n* = 700) of various psychological therapies for tinnitus, which converged on there being a large and sustained positive effect on tinnitus annoyance; CBT was demonstrated to be more effective than the other psychological therapies examined. Smaller effects were obtained for measures of negative affect and sleep problems. Hesser et al. (75) looked at 15 randomized controlled trials (*n* = 1091) and found CBT to have a significant positive impact on tinnitus-related distress. The Cochrane review of CBT for tinnitus (17, 76) used change in tinnitus loudness (self-reported on a numeric scale) as the primary outcome, and change in tinnitus-related quality of life, depression, or mood as secondary outcomes. The review found no effect of CBT on self-reported loudness but did find an improvement in tinnitus-related quality of life when CBT was compared to other interventions or no treatment, and a small improvement in depression when CBT was compared to no treatment. The use of self-reported loudness as the primary outcome measure restricted inclusion in the review as many CBT studies do not measure it.

A more recent randomized controlled trial (39) reported the effects of specialized CBT that is informed by the fear-avoidance model, used successfully in the treatment of chronic pain (77). Compared to controls, the intervention group reported a greater improvement in both tinnitus severity (TQ score) and depression and anxiety (measured by the global score on the HADS) at 12 months follow-up. Scores for anxiety and depression were not reported separately, and so further research is required to provide a more complete picture.

#### Connections to other components of the tinnitus model

While work by Cima et al. (32) suggests that fear is associated with greater attention to tinnitus, this potential link between emotional distress and selective attention has not been directly assessed. The cognitive model makes the specific prediction that arousal and emotional distress lead to selective attention and monitoring.

### Selective attention and monitoring

It has been noted that when people are psychologically distressed, they focus more on the cause of this distress or on the experience itself (78). From a clinical perspective, this component of the model can be regarded as the result of thinking about tinnitus in threatening terms and of the ensuing increase in arousal. The consequence of such thinking, and increased arousal, is to increase the perception of tinnitus. If this increases the perceived threat of tinnitus, then a maintenance cycle will be established. The precise process underpinning this component of the model still needs further consideration, but two theoretical concepts within the attentional field might explain this phenomenon (Figure 1). Selective attention describes a process by which an individual orients toward a specific stimulus within the external or internal environments, while suppressing irrelevant or competing stimuli. This is a central concept within several theoretical models of attention and explains how the cognitive system resolves its limited processing capacity (79, 80). Monitoring indicates a more sustained orienting process, perhaps motivated by a hypervigilant state (81). Maintaining concentrated attention over prolonged periods of time toward a particular stimulus is encapsulated within theoretical models as “sustained attention” (80).

#### Selective attention and monitoring in tinnitus

A number of studies indicate that people with tinnitus are more impaired on tasks that place demands on specific aspects of attentional processing, than are people without tinnitus (82–85). The effect of tinnitus on sustained attention is very poorly documented. A few studies have investigated this (83, 84, 86), but did not show any systematic difference between tinnitus and hearing impaired groups. A greater number of studies have addressed selective attention, although not always in the auditory modality.

Because it is not possible to directly measure selective attention, it is typically investigated by contrasting performance (usually reaction times) on pairs of carefully controlled stimulus conditions. One of the early studies on tinnitus and selective attention merely reported absolute reaction times (87) and so interpretation of results is limited. Cuny et al. (88) used a modified version of the dichotic listening paradigm to compare performance in the tinnitus versus the non-tinnitus ear. They required individuals to listen to two tones played sequentially to the two ears and judge the relative pitch of the tones. Accuracy was worse for tones presented to the non-tinnitus ear than the tinnitus ear, perhaps suggesting a difficulty in orienting selective attention away from the task-irrelevant sound (i.e., the tinnitus). However, an opposite effect was found for subjects with severe tinnitus symptoms, making it difficult to reconcile this finding with the previous interpretation. A more recent well-controlled study has assessed selective attention using the Attention Network Test (85), a well-validated visual test that measures selective attention by the performance difference between a condition with a valid spatial cue and one without a spatial cue. Results from this study did point to an attentional deficit among tinnitus subjects but one that suggested a difficulty in the executive control of attention (i.e., the ability to focus on task relevant information and inhibit processing of irrelevant information) rather than in the selective attention component. Interestingly, a recent study by Hoare et al. (89) also found no correlation between tinnitus handicap and performance on a visual version of a selective attention task as measured by The Test of Everyday Attention (80). However, as these studies assessed selective attention in the visual domain, they would not have been sensitive to impairments in the auditory modality. There is some evidence that modality differences exist on this test of selective attention (90). Overall, the evidence available does point to some sort of attention-related difficulty among people troubled by tinnitus. The amount of evidence is as yet very limited and this is certainly an area where further study is needed.

A question relevant to the cognitive model concerns the role of selective and sustained attention in everyday life. Experimental studies do not assess this directly, and there has been little investigation of everyday life situations. In the clinical setting, patients tend to report that tinnitus makes it more difficult to focus their attention, especially during conversation (91). For example, Hiller and Goebel (87) found a high correlation between annoyance and self-reported inability to ignore tinnitus among a subgroup of “highly annoyed” subjects. Two further clinical surveys indicate that patients seeking tinnitus treatment reported being aware of their tinnitus about two-thirds of the waking day (92, 93), and 79% of people responding to a survey by the German Tinnitus Association reported being aware of tinnitus “all the time” (69). A caveat for interpreting these data is that selective attention and awareness do not necessarily define the same theoretical construct.

No other studies to date have investigated everyday monitoring behavior among patients with tinnitus, although a number of case studies [e.g., Ref. (94)] have given examples of the kind of monitoring behavior engaged in by patients such as consciously listening to and self-rating tinnitus loudness in different environments or after different activities. A study by Cima et al. (32) adapted the pain vigilance and awareness questionnaire (77) for use with patients with tinnitus. This questionnaire includes items that fit the definition of selective attention (inability to ignore tinnitus, dominance of tinnitus over other things), as well as overt monitoring behavior. The authors found a negative correlation between scores on this questionnaire and a quality of life measure, indicating that selective attention and monitoring may indeed be part of a more negative tinnitus experience.

#### Effect of tinnitus interventions on selective attention and monitoring

A novel intervention that works on attention shifting using music has recently been trialed by Pape et al. (95). They found significant neuroplastic changes post-training using magnetoencephalography but they did not measure participants’ perception of whether their attention to tinnitus had altered. There was no significant change in tinnitus handicap, but baseline THI scores were relatively low. Hoare et al. (89) administered a number of frequency discrimination training auditory games designed to interrupt tinnitus, possibly by diverting attention away from the tinnitus sound and toward an externalized sound source. However, the training had no impact on performance on an auditory sustained attention task as measured by The Test of Everyday Attention (80). Other studies have included “attention shifting” exercises as part of broader tinnitus rehabilitation (37, 39, 96). Although all of these report reductions in tinnitus distress post-therapy, none report specifically on whether ability to shift attention from tinnitus improved, and so it is unclear whether reduced attention deficits are a key component of reduced distress.

#### Connections to other components of the tinnitus model

Cima et al. (32) reported a significant positive correlation between scores on their novel Tinnitus Vigilance and Awareness Questionnaire (TVAQ) and on their catastrophizing and tinnitus-related fear questionnaires, suggesting a possible link between negative automatic thoughts, beliefs, and tinnitus monitoring. However, it has not been formally established whether this questionnaire measures the same theoretical construct as sustained attention.

### Distorted perception

For some conditions, the process of selective attention and monitoring is considered to be a contributory factor in distorting perception. For example, Harvey (97) and Harvey and Tang (98) suggest that distorted perception of sleep parameters is ubiquitous among people suffering from insomnia and attribute this to selective attention.

#### Distorted perception in tinnitus

If our model is correct, then patients with tinnitus should also exhibit a distorted perception of their tinnitus. Several different perceptual attributes could be plausible candidates for distortion including timbre, pitch, and loudness. The possibility that perception may be distorted was first mooted by Fowler (99), who suggested that patients with tinnitus “experience an exaggerated sensation as to both its loudness and its timbre, and it is then overestimated and sensed as a most disagreeable or unbearable noise” (p. 396). Since then, however, the subject has been debated only with respect to loudness. Given the available evidence, we will therefore restrict our discussion to this focus.

##### Is loudness perception distorted?

If people’s perception of tinnitus loudness is distorted, we might expect to find a mismatch between a psychoacoustic estimate determined by matching the loudness of an external tone to tinnitus loudness, and self-reported loudness measured on a rating scale. Self-reported, tinnitus may be rated as extremely loud, where a psychoacoustic measure would suggest otherwise. An approach to grading self-reported loudness developed by Klockhoff and Lindblom (100) involves classifying tinnitus as either Grade I; tinnitus is described as audible only in a silent environment, Grade II; tinnitus is audible in ordinary acoustic environments but is masked by loud environmental sounds, or Grade III; tinnitus is audible in all acoustic environments. Andersson et al. (4) reported that 4–5% of their study population could be classified as having Grade I tinnitus; 57–64% as Grade II; and 31–38% as Grade III. The implication is that the majority of tinnitus clinic patients describe their tinnitus as loud enough to compete with strong environmental noises. Baguley et al. (101) note that patients with tinnitus commonly compare their internal noises to external sounds, such as a loud cutting tone, screams, or loud bag-pipes.

These observations that tinnitus is perceived as a loud stimulus have to be reconciled with the psychophysical measurement of tinnitus. The proxy measure of loudness most commonly involves matching to a reference tone. There is a convention of expressing the estimate in terms of decibels sensation level (dB SL), representing the sound level above the hearing threshold. The literature regarding loudness matching of tinnitus was reviewed by Tyler (102) who noted that a match of ≤10 dB SL was consistently found across studies. For people with either a mild or no hearing loss, this measure corresponds to a very low intensity sound. A number of methodological difficulties arise with this procedure. First, estimates of loudness of tinnitus vary when subjects are retested (103, 104). This may reflect unreliability in the measurement procedures or variation in the tinnitus from day to day. Second, many patients are unable to match their tinnitus to an external sound (5, 105). Third, it has been argued that measuring tinnitus in terms of dB SL may be inappropriate as it neglects psychoacoustical factors such as recruitment (i.e., non-linear loudness growth) (102) and it assumes that tinnitus can be regarded as any external sound. Various attempts have been made to compensate for this such as measuring loudness in sones (106) or matching to a low frequency tone (107), but there is no consensus. While acknowledging the criticisms of the tinnitus matching procedure, loudness match of tinnitus seems to be typically of low intensity and at best weakly correlates with tinnitus handicap (108, 109). This suggests that people’s self-reports overestimate the “true” loudness of tinnitus as measured by conventional matching techniques.

##### The relationship between loudness and distress

The cognitive model proposes that distorted perception contributes to tinnitus distress. Whereas most studies report weak correlations between tinnitus distress and psychoacoustic loudness [e.g., Ref. (105, 110–112)], self-reported loudness and tinnitus distress moderately correlate. For example, Wallhausser-Franke et al. (69) asked more than 4000 members of the German Tinnitus Association to rate their tinnitus loudness on a scale of 1–10 and their tinnitus distress on the brief version of the German TQ, and found a moderate correlation between the two, concluding that self-reported loudness and distress represent and should be assessed as two different constructs. In an earlier study of nearly 5000 members of the same association, Hiller and Goebel (87) again found that Klockhoff and Lindblom grading and TQ scores only moderately correlated. Kuk et al. (31) and Weise et al. (35) also found moderate correlations between scores on a tinnitus handicap measure and loudness self-rated on a VAS. In general, therefore, self-reported loudness scales measure a construct that is different from those measured by either tinnitus distress questionnaires or the psychoacoustic estimates of tinnitus loudness matched to external tones. Consistent reports of moderate correlation between self-reported loudness and tinnitus distress suggest some relationship between the two measures.

#### Effects of tinnitus interventions on loudness

If perceived loudness is a function of distorted perception in distressed individuals, we would expect to see it reduce to coincide with reduced distress. However, many intervention studies illustrate that distress changes post-therapy while self-rated loudness stays the same. In their meta-analysis of psychological therapies for tinnitus, Andersson and Lyttkens (74) noted weak effects on tinnitus loudness but significant effects on tinnitus-related distress. In their review of CBT for tinnitus, Martinez-Devesa et al. (17) also conclude that self-reported loudness does not change as a result of therapy, while tinnitus-related quality of life moderately or substantially improves. There is, however, a problem inherent in interpreting self-perceived loudness, in that it is unclear exactly what construct is being measured. Furthermore, as a tool, it cannot easily be uncoupled from the influence of selective attention. All the studies included in the reviews mentioned above asked people to make a single subjective rating of the loudness of their tinnitus at specific time points (some of them daily, others just once every few weeks). Clinical impressions tell us that even when distress is much reduced, most people perceive tinnitus as prominent when invited to listen to it and rate its loudness, in the same way that most people perceive a ticking clock as loud if asked to pay special attention to it. If therapy reduces a patient’s negative thoughts about tinnitus, then there will be less arousal and less distress and this in turn will lead to less selective attention and monitoring. Tinnitus will, therefore, be less prominent in the person’s life as the person will attend to it less. Asking a person to deliberately attend to and rate tinnitus would be expected to reverse at least the latter part of this process and, therefore, influence the ratings of loudness.

#### Connections to other components of the tinnitus model

It is hypothesized that overestimation results from selective attention and monitoring of tinnitus. Both automatic and deliberate behaviors are at work here. Just as Harvey and Schmidt (113) observed that monitoring the clock led people to make longer estimates of sleep latency, so a similar process may be operating for tinnitus. Active monitoring may also increase the chance of detecting random changes in tinnitus or in the signal/detection properties of tinnitus that are not intrinsically significant but to which the patient might attribute *overly* negative meaning. The cognitive model predicts more specifically that distorted perception leads to an increase in negative automatic thoughts. Some support for a connection between loudness and negative thinking is provided by Weise et al. (35) who found a moderate but significant correlation between catastrophizing and self-rated tinnitus loudness. It is proposed that negative evaluation, and in particular negative images of tinnitus (e.g., it sounds like a drill; it sounds unnatural) also lead to overestimation. Further research into this is warranted.

#### Beliefs in tinnitus

This variable in the model is certainly congruent with a cognitive therapy approach but there is as yet little direct research evidence to inform the matter. Illness representations in patients with tinnitus have been investigated in only a small number of studies. They have been found to be associated with the extent of tinnitus-related complaint as measured by the TQ (114). Positive illness representations have been found to be associated with reduced levels of depression in patients with tinnitus (115–118). Different studies have identified different illness representations as particularly influential in mediating this relationship in patients with tinnitus. Vollmann et al. (118) reported that dispositional optimism is associated with more positive illness representations. They found that optimists compared with pessimists perceived their tinnitus as associated with fewer symptoms, a less chronic timeline, less serious consequences, and as more controllable and understandable. They concluded that more favorable illness representations mediated the relationship between optimism and depression in patients with tinnitus. Similarly, Andersson (115) also found that the trait of optimism is negatively associated with tinnitus distress.

While there is little research specifically on beliefs relating to tinnitus, there is a larger body of research about personality factors in people with tinnitus. While the concepts of personality and beliefs will not precisely overlap, the findings from personality research may allow some general inferences about the way people with tinnitus see the world; such factors are said to modulate illness representations (119). As might be expected, patients with tinnitus as a wider group have been found to have normal personality profiles (120–122). However, some studies have supported the idea that greater tinnitus distress is associated with specific personality traits measured on the Minnesota Multiphasic Personality Inventory (MMPI) (123) and the Eysenck Personality Quotient (EPQ) (124). For example, the MMPI traits of depression (121) hysteria and hypochondriasis (125), lower levels of the EPQ measure of extraversion (126), and higher levels of neuroticism (127) have all been associated with higher tinnitus distress. Other studies have also pointed to an association between tinnitus severity and traits of perfectionism (128) and anxiety sensitivity (47, 129); traits that contain within them beliefs about how things should be and the meaning of symptoms. The issue is further informed by the work of Bartels et al. (130), who examined the relationship between Type D personality and tinnitus distress. Type D personality is characterized by high levels of negative emotion (generally sad and gloomy beliefs about life) and social inhibition that means the person does not tell others about their emotions. They reported that patients with tinnitus with Type D personality had greater psychological distress and poorer health-related quality of life than non-Type D patients.

The topic of beliefs is also informed by the literature on personal control. The concept of “locus of control” refers to the tendency a person has to explain events as caused by internal (within the person’s responsibility) or external (outside the person’s responsibility) factors. Personal control was found to be an important predictor of tinnitus discomfort and of adaptation in a study by Scott et al. (131). A significant relationship between locus of control, tinnitus severity, and emotional distress in tinnitus sufferers was also demonstrated by Budd and Pugh (132). In a study of the psychological profile of patients with tinnitus, Attias et al. (110) found that patients who sought help for tinnitus had a greater external locus of control than those who did not seek help for tinnitus. One study reported on an internet survey of more general health-related beliefs among patients with tinnitus (133). Holding positive beliefs about being able to control one’s own health was associated with less depression and a greater sense of well-being in people with tinnitus. Beliefs about control predicted adjustment irrespective of the severity of tinnitus, suggesting that it is not just that people with milder tinnitus are better able to maintain beliefs about personal control.

These studies seek to assess stable personality characteristics rather than temporary emotional states. The influence of long-term characteristics is evidenced by a study by Langenbach et al. (73). These researchers examined the personality profile of people within weeks (mean time of 11 days) of the onset of tinnitus. They reported that the presence of anxiety and dissatisfaction with life at that time predicted later tinnitus distress, while psychophysical characteristics of tinnitus and other concomitant physical symptoms did not. They argued that as the psychological factors being measured were present very close to the onset of tinnitus, they are stable traits likely to have been present before the onset of tinnitus.

Collectively, these findings strongly suggest that people who are prone to hold more negative beliefs about themselves and the world are likely to experience tinnitus as more distressing. This view is reflected in the literature on specific tinnitus-related beliefs or illness representations. The body of evidence can be taken as supportive of the idea that beliefs are a factor in the experience of tinnitus.

#### Effects of tinnitus interventions on beliefs

To date, no studies have specifically reported on the effect of intervention on beliefs. Altering beliefs is likely to be a factor contributing to the success of CBT. None of the outcome studies on the use of CBT in tinnitus management explicitly target beliefs. It is, however, possible that CBT that addresses negative automatic thoughts and accompanying behaviors can alter more general beliefs. Whether or not beliefs need to be altered remains untested.

#### Connections to other components of the tinnitus model

General factors such as personality traits are said to influence the development of specific beliefs such as those about health and in turn specific health conditions and specific thoughts. This is in keeping with the model proposed here. Vollmann et al. (114) reported that the effects of some illness representations in patients with tinnitus were partially mediated by the use of negative self-statements, and others were fully mediated by those statements. Similarly, Sirois et al. (133) argued that the sense of control discussed in their study had an effect through influencing the meaning or significance of tinnitus rather than directly on tinnitus *per se*. They acknowledge that the cross-sectional nature of their study meant that the direction of causality between severity, control, and adjustment indicators could not be established. They, nonetheless, argue that the theoretical underpinnings of cognitive adaptation theory make the causal order (*viz*. beliefs about control leading to an interpretation of tinnitus that influences adjustment to the symptom) plausible. These findings are congruent with our cognitive model. Evidence from other studies also suggests the relationship between personality variables and distress is not direct but instead is mediated by cognitive variables such as dysfunctional thoughts (134), particularly catastrophizing (35).

### Safety behaviors

Safety behavior refers to actions that people take to avoid the feared consequences of a particular event (135). Central to the understanding of safety behavior is the argument that the behavior is a function of the cognitive evaluation of the event. If an event is interpreted *overly* negatively, i.e., the thoughts contain cognitive distortions, then the actions the person takes to rectify the situation or prevent the perceived threat from materializing may prevent the person from realizing that the thoughts are *overly* negative. The behavior, therefore, prevents the thoughts from being disconfirmed. The behaviors can be clear overt actions that any observer could witness or may be covert “mental” actions such as trying not to think about certain things.

#### Safety behavior in tinnitus

Although clinical experience suggests that safety behaviors are commonly used by patients with tinnitus, they have not been as well researched as for patients with other conditions such as insomnia. The use of coping behavior has been investigated in a number of studies; these behaviors are perhaps the nearest thing to safety behaviors in the literature. A helpful distinction between the everyday and scientific use of the word “coping” is given by Andersson et al. (136).While in everyday use the word refers to *effectively* dealing with tasks, the scientific use is independent of the outcome and includes behavior that has negative consequences. A common example of the latter is avoidance coping.

There is some indication that use of avoidance behavior is linked to more distressing tinnitus; it has been found to be a significant predictor of tinnitus distress (47). An interview study (4) found that 62% of 216 tinnitus clinic patients reported “avoidance of situations” due to tinnitus. Examples are not given in this study, but McKenna and Irwin (137) observe a common practice is avoidance of silence due to fear of being unable to cope if fully exposed to tinnitus for even a short while. A study using a revised version of the Ways of Coping Questionnaire also found that avoidant coping was associated with tinnitus disability (138). The Tinnitus Coping Strategy Questionnaire (139) was used to study this issue in an internet survey of Swedish people with tinnitus (136). A greater use of coping strategies was found to be associated with more distress. The correlation between the benefits derived from the coping strategies and tinnitus distress was low, suggesting that the observations did not simply reflect more distressed people trying more coping strategies than people with less annoying tinnitus. Men with severe tinnitus have been found to more often engage in “escape coping” (e.g., wishful thinking, taking drugs or alcohol to feel better) than men with milder tinnitus or no tinnitus (140). This study, however, assessed general strategies for coping with stress rather than specific strategies for coping with tinnitus. Budd and Pugh (40) constructed a questionnaire to investigate coping styles among patients with tinnitus that included items about avoiding situations along with behaviors like complaining to others and wishing tinnitus away. They found that “maladaptive coping” correlated with severity of tinnitus but they did not examine avoidance as a distinct component of maladaptive coping. A Tinnitus Fear-Avoidance Scale (TFAS) was developed by Kleinstauber et al. (64) that measures fears about tinnitus and avoidance behavior related to these. Using the THI, they divided their patients into five categories according to severity of tinnitus handicap and found a significant effect of category on TFAS scores, suggesting that people who use avoidance behavior and have fearful beliefs are more likely to be troubled by tinnitus. However, the relative importance of beliefs and behavior cannot be ascertained. Although many of these studies have not fully separated avoidance behavior from other constructs, taken together this literature points to the importance of behavior in tinnitus distress and implies that behaviors that maintain negative thoughts are particularly important.

#### Effects of tinnitus interventions on safety behavior

It is likely that some interventions based on “manualized” forms of therapy will result in patients making behavioral changes (e.g., avoiding silence, maintaining distraction) that inadvertently maintain their fears. Within a CBT setting, people with tinnitus are often encouraged to abandon their safety behaviors by carrying out “behavioral experiments” such as increasing or decreasing environmental noise levels and noticing the effect on tinnitus and how they feel (94). Although behavior change is described as an important component of successful CBT programs (39), changes in behavior have not yet been measured separately from thoughts and emotions in tinnitus intervention studies. As it is possible to support patients in conducting behavioral experiments outside the context of a full CBT program, ascertaining the effectiveness of this kind of intervention seems particularly important.

#### Connections to other components of the tinnitus model

A link between safety behavior, negative thoughts and beliefs has been proposed by McKenna (141) and by Cima et al. (32), but there is no empirical evidence to fully support these ideas at present. Both Kleinstauber et al. (64) and Hesser and Andersson (47) have measured anxiety sensitivity. Although this concept *per se* is not included in the cognitive model, it does contain within it an element of catastrophic thinking (e.g., “my heart is thumping so hard I’m going to collapse”). These researchers demonstrated that avoidance behavior partially mediates between anxiety sensitivity and tinnitus distress (47) or tinnitus catastrophizing (64), which suggests that avoidance behavior may play a part in intensifying negative thought.

### Causality

It can be seen from the discussion above that, while there is a reasonable body of evidence to support most of the individual components of the model, there is less evidence supporting the proposed links between individual components. Moreover, most of the work investigating links between components is correlational, and so it is not possible to draw conclusions about causality. It is unlikely that causal relationships are straightforward. When questioned, many people who are troubled by tinnitus also acknowledge other sources of distress in their lives (49, 56, 57), which may pre-date the tinnitus, so pre-existing emotional distress may contribute to negative thinking about tinnitus as much as negative thinking contributes to emotional distress. Indeed, the aforementioned investigation of patients who had very recently developed tinnitus (73) found that those with anxiety at the time of onset were more likely to be severely distressed by their tinnitus 6 months later. However, other work (142) has noted the development of depressive symptoms some months *after* the onset of tinnitus.

While acknowledging that feelings about other life experiences may well influence people’s reactions to tinnitus, we propose that, when tinnitus develops, causal relationships are broadly as described in Figure 1. This is in keeping with comparable cognitive models relating to insomnia (26) and chronic pain (77) and with the theory underpinning CBT (27), which is built around the idea that negative thoughts give rise to negative emotions.

Given that our model consists of a series of interlinked testable hypotheses, it would lend itself to testing with structural equation modeling. This technique has been developed explicitly to test both goodness-of-fit of a theoretical model to observed data and to test the validity of causal structures (143). While certainty over causation is rarely possible and causation is rarely unidirectional, the validity and strength of our proposed causal relationships can be empirically tested.

## Implications for Cognitive-Behavioral Therapy

The proposed model gives a more prominent role to cognition than previous psychological models, particularly in understanding behavioral changes in cognitive terms.

The implications of this proposed model are that psychological therapy should focus on each of the following activities:

• correcting negative automatic thoughts,
• reducing sympathetic autonomic nervous system activity,
• reducing selective attention and monitoring for tinnitus-related cues,
• correcting distorted perceptions of tinnitus intensity and its impact on functioning,
• correcting counterproductive safety behaviors, and
• correcting inaccurate beliefs.

Conscious cognitive processes are seen as having a central role in our model. Like other models, we recognize some possible roles of automatic processes but we see the therapeutic targets as primarily conscious cognitive ones. In a careful consideration of the interaction between conscious and automatic processing, Kahneman (20) argues that automatic processing errors can be corrected only via conscious cognitive processes. The targeting of conscious cognitive processes is, therefore, consistent with modern theorizing. Cognitive therapy aimed at identifying and changing negative automatic thoughts in patients with tinnitus has already met with some success (17, 75). Our proposal allows these successes to be interpreted within a coherent model. It is to be hoped, however, that further improvement in outcomes can be made. A clearer understanding of the role of thoughts in the overall process would help this.

The present status of techniques for modifying arousal is one in which “a healthy eclecticism holds sway” (144). A variety of strategies have been found helpful for some patients at least. Overall, gains have been modest and not always long lasting. While Jastreboff (11) states that relaxation training cannot be an effective strategy in tinnitus management, he does not give his reasons for this assertion. While there is enough evidence to suggest that relaxation training be regarded as a solid standby [see Ref. (16, 101) for reviews], its modest successes may result from insufficient consideration being given to cognitive processes.

A creative approach to reducing selective attention and monitoring of tinnitus is needed. To date, therapy strategies have focused on the manipulation of environmental sound levels to alter the signal-to-noise properties of tinnitus and so reduce selective attention and monitoring of tinnitus (145). Techniques for switching attention have been explored (37, 146) but the findings are inconsistent and more work is needed in this area. Any given task is likely to be associated with a decline in its ability to be engaging over time, and a variety of such tasks may need to be developed.

Techniques for correcting distorted perception and imagery of tinnitus need to be developed. It is no longer customary to carry out psychoacoustic tinnitus loudness and pitch balancing in a clinical setting as this is thought to add little to the diagnosis or management. For this reason, clinicians tend to pay little attention to patients’ descriptions of their tinnitus. The treatment of patients with tinnitus might be improved by attending to this information and trying to correct distortions in it. Andersson and Kaldo (147) suggest that “it is important to work toward acceptance of tinnitus and to foster the idea that tinnitus is not worth the attention it gets” (p. 99). This would seem to be a difficult thing to achieve if tinnitus really is, or the patient continues to believe that tinnitus is, of the intensity of, say, a jet engine. No formal evaluations of techniques that seek to change distorted perception, or tinnitus imagery, have yet been carried out. It is likely that this will again involve the targeting of conscious evaluations of the tinnitus signal.

The notion of safety behaviors allows the model to accommodate seemingly opposite behavioral responses, particularly the use of environmental sound and the avoidance of sound. If the conceptualization of tinnitus proposed here is correct, then the standard TRT recommendation of a sound enriched environment for all patients is likely to maintain or worsen the problem for some patients. Placing behavior within a cognitive context will help to avoid this.

The model proposed here suggests that cognitive therapy should also address beliefs in patients with tinnitus. Hallam and McKenna (144) describe cognitive therapy with a patient who holds unhelpful assumptions and beliefs; this is the only example of this type of work that the authors are aware of. The psychoeducation that is often given to patients in a clinical setting may represent a crude attempt to address beliefs. This is an area where more work is needed; this may be informed by the emerging literature on illness representations.

## Conclusion

A cognitive model of tinnitus is proposed here that is in keeping with recent theorizing in clinical psychology. It is suggested that the key components maintaining tinnitus distress are negative appraisal of tinnitus, arousal and distress, selective attention and monitoring, erroneous beliefs, counterproductive safety behaviors, and a distorted perception of tinnitus. This differs from the previous psychological perspectives, in that there is a greater emphasis on cognition and in particular it sets behavioral responses to tinnitus in a cognitive context. The assertion that patients with tinnitus have a distorted perception of their noise, although suggested by Fowler in 1942, is also at odds with the views of some other current authorities [see Ref. (101) for a review] and the suggestion that this distortion is influenced by cognition is also new. Highlighting the role of beliefs in the tinnitus experience is something that has not been done before (other than by Hallam and McKenna) (144).

The evidence reviewed here provides even greater (although not unequivocal) support for a psychological model of tinnitus distress than was possible when Hallam et al. (7) first proposed such a model. However, as yet, there is only a modest amount of robust research evidence to support some components of the model proposed here. In particular, the hypothesized role of safety behaviors, beliefs, and distorted perception need to be further investigated. The proposed conceptualization of tinnitus gives rise to a number of testable hypotheses. It is to be hoped that further development of the model might provide the psychological perspective with a firmer scientific basis and may lead to the development of a more successful approach to therapy.

## Conflict of Interest Statement

The authors declare that the research was conducted in the absence of any commercial or financial relationships that could be construed as a potential conflict of interest.
